# The value of the fore-sight^TM^ monitor in the postoperative phase after congenital cardiac surgery. a descriptive statistical interim-analysis

**DOI:** 10.1186/2197-425X-3-S1-A595

**Published:** 2015-10-01

**Authors:** HGJ Delrue, F Guiza, D Vlasselaers, T Fivez, L Desmet, G Van den Berghe, G Meyfroidt

**Affiliations:** Intensive Care, KU Leuven, Leuven, Belgium

## Introduction

The FORE-SIGHT^TM^ monitor measures absolute cerebral tissue oxygen saturation (S_ct_O_2_) of the frontal lobes of the brain, in a non-invasive way, through near-infrared spectroscopy (NIRS) [1]. Its value for early detection of hemodynamic deterioration in the postoperative phase after congenital cardiac surgery has never been examined. In a prospective observational study [2], 300 mechanically ventilated patients younger than 12 years after cardiac surgery will be monitored with NIRS, in addition to their routine haemodynamic monitoring. The NIRS monitor is blinded to clinicians, and its predictive value to detect predefined critical hemodynamic events will be analysed.

## Objectives

To compare S_ct_O_2_ in relationship to the arterial oxygen saturation (S_a_O_2_) and central venous oxygen saturations (S_cv_O_2_), in children with cyanotic (CC) and non-cyanotic cardiopathy (NCC) admitted to the paediatric intensive care unit (PICU) after congenital cardiac surgery.

## Methods

The present study is a preliminary report of baseline data and monitored values of the first 136 children included in the abovementioned prospective observational FORE-SIGHT^TM^ study.

## Results

The results are summarized in table 1 and table 2. 83 children were admitted with a CC, 53 with NCC, and 57.3% were boys. Patients in the CC-subgroup had a longer median PICU length of stay, had lower mean arterial blood pressures, and higher haemoglobin levels compared to patients in the NCC-subgroup. Evidently, CC-patients had a lower mean S_a_O_2_. There was a non-significant trend towards lower S_cv_O_2_ and S_ct_O_2_ values in the CC subgroup (which was significant for L- S_ct_O_2_). S_cv_O_2_ was significantly lower than S_ct_O_2_ in the CC-subgroup (p=0.002), but not in the NCC-subgroup (p=0.28).

**Table 1:** Table 1

	Overall	Cyanogenic	Non-Cyanogenic	p-value (*)
Number (boy/girl ratio)	136 (57.3%)	83 (56.6%)	53 (58.5%)	
Age in months: median (IQR)	5.5 (2-18.5)	5 (2-19.8)	6 (2-17)	0.61
PICU LOS in days: median (IQR)	5 (3-9)	6 (4-12)	3 (2-6)	0.0004
Hemoglobin (g/dl)	10.9 (1.2)	11.1 (1.2)	10.5 (1.1)	0.005
Mean arterial pressure (mmHg)	63.7 (9.6)	61.7 (9.5)	66.8 (9.1)	0.003
Mean and SD was reported, except for age and ICU LOS. (*) P-value of the comparison of the cyanogenic and non-cyanogenic groups. T-test was used to compare means, Wilcoxon ranking-test for medians.

**Table 2:** Table 2

	Overall	Cyanogenic	Non-Cyanogenic	p-value (*)
SaO2 (%)	92.7 (7.4)	90.7 (8.0)	95.7 (4.9)	< <0.0001
ScvO2 (%)	67.3 (12.7)	65.5 (11.9)	70.8 (13.7)	0.07
L-SctO2 (%)	71.6 (7.2)	70.6 (7.8)	73.1 (5.9)	0.04
R-SctO2 (%)	71.3 (6.9)	70.5 (7.7)	72.6 (5.2)	0.08
A-SctO2 (%)	71.7 (6.5)	70.8 (7.3)	73.1 (4.8)	0.06
ScvO2 < A-SctO2		p=0.002	p = 0.28	
Mean and SD was reported, except for age and ICU LOS. (*) P-value of the comparison of the cyanogenic and non-cyanogenic groups. T-test was used to compare means, Wilcoxon ranking-test for medians.				

## Conclusions

Notwithstanding the lower observed S_a_O_2_, CC patients are able to preserve brain tissue oxygenation as measured by NIRS, which is significantly higher than their S_cv_O_2_. The additional predictive value of NIRS in this setting will be assessed in the ongoing Fore-sight study.

## Trial Registration

Clinical trial registered with www.clinicaltrials.gov (NCT01706497).

## Grant Acknowledgment

Foundation for Scientific Research Flanders (FWO) (G. 0904.11). Senior clinical investigator, FWO to Geert Meyfroidt (1846113N). Methusalem program, Flemish Government to Greet Van den Berghe (METH/08/07).
